# A Method to Determine the Efficacy of a Commercial Phage Preparation against Uropathogens in Urine and Artificial Urine Determined by Isothermal Microcalorimetry

**DOI:** 10.3390/microorganisms10050845

**Published:** 2022-04-20

**Authors:** Aurelia Pahnita Sigg, Max Mariotti, Anabel E. Grütter, Tecla Lafranca, Lorenz Leitner, Gernot Bonkat, Olivier Braissant

**Affiliations:** 1 Department of Biomedical Engineering, University of Basel, Gewerbestrasse 14, 4123 Allschwil, Switzerland; aureliapahnita.sigg@stud.unibas.ch (A.P.S.); max.mariotti96@gmail.com (M.M.); anabel.gruetter@stud.unibas.ch (A.E.G.); tecla.lafranca@stud.unibas.ch (T.L.); 2 Department of Neuro-Urology, Balgrist University Hospital, University of Zürich, 8008 Zürich, Switzerland; lorenz.leitner@balgrist.ch; 3 Alta uro AG, Centralbahnplatz 6, 4051 Basel, Switzerland; bonkat@alta-uro.com

**Keywords:** isothermal microcalorimetry, bacteriophages, phage cocktail, *E. coli*, *P. mirabilis*

## Abstract

Background: Urinary tract infections are commonly encountered and often treated with antibiotics. However, the inappropriate use of the latter has led to the appearance of resistant strains. In this context we investigate the use of calorimetry to rapidly determine if a phage cocktail can be used as alternative to antibiotics. Methods: We used a commercially available phage cocktail from an online pharmacy and tested it against a strain of *Escherichia coli* and a strain of *Proteus mirabilis*. We used isothermal microcalorimetry to follow the metabolic activity of the bacterial culture treated with the phage cocktail. Results: Isothermal microcalorimetry was able to follow the dynamic of the bacterial metabolic activity reduction by the phage cocktail. Both pathogens were strongly inhibited; however, some regrowth was observed for *E. coli* in urine. Conclusions: Isothermal microcalorimetry proved to be a valuable technique when investigating the efficacy of phage cocktails against uropathogens. We foresee that isothermal microcalorimetry could be used to obtain rapid phagograms.

## 1. Introduction

Urinary tract infections (UTI) are the second most common infectious diseases diagnosed after respiratory infections [1,2]. They can affect the entire urinary tract and progress toward urosepsis and septic shock, a life-threatening condition [3]. The most common uropathogen that causes between 80 and 85% of UTIs is *Escherichia coli* [4,5], a gram-negative, facultative anaerobic bacterium that is part of the normal human gut microbiota. Still, some strains can be virulent and are responsible for a wide range of diseases that have an intestinal or an extra-intestinal origin [6,7]. Another commonly encountered pathogen of the urinary tract that is responsible for up to 10% of all UTIs is *Proteus mirabilis*. *P. mirabilis* is another gram-negative rod-shaped bacterium, usually encountered in complicated UTIs (e.g., catheter-associated UTI and patients with abnormalities of the lower urinary tract) and often associated with phosphate stone formation due to its urease production [8,9]. In catheter-associated UTI, this can lead to crystal formation (mostly struvite-NH_4_MgPO_4_·6H_2_O) and ultimately to catheter blockage.

The extensive and sometimes inappropriate use of antibiotics in the previous years has led to the rise of antimicrobial resistance in both *E. coli* and *P. mirabilis*. Recent reports [10,11] on resistance rates to commonly used antibiotics show proportions of resistant isolates between 39.7% and 98% for *E. coli* [10,11] and 21% and 91% for *P. mirabilis* with variations depending on the geographic location. Finding an alternative to fight resistant strains of these pathogens is therefore highly needed [9,12,13]. This is even more important considering the interplay and correlations between the resistance observed [14,15].

Among other alternatives, the therapeutic use of bacteriophages (phages) is receiving more attention [16,17]. These naturally existing viruses specifically kill bacteria, and were first described by Félix D’Hérelle, who observed their antibacterial activity. Phages are highly specific to one bacterial species or strain and do not infect eukaryotic cells making them a valuable alternative to antibiotics [16,18]. Indeed, in 1919, Félix D’Hérelle successfully applied phages to treat bacterial infections [16,17]. However, the discovery of penicillin and other wide spectrum antimicrobials, as well as the inconsistent results of phage therapy in early clinical trials diminished the interests in phages having a much narrower spectrum of activity [19,20]. The research and use of phage thus rapidly decreased, except in eastern European and post-Soviet countries, in which phage therapy is well accepted and where commercial products are available. Nowadays, an increase in antimicrobial resistance has renewed the interest in phage therapy to treat bacterial infections, including UTI [21,22,23].

As phages have a species-specific host range and can penetrate biofilms, one can use their abilities to stop the spread of resistant pathogens [20,24]. However, the drawback of the narrow host range is the necessity to find the right phage for the right infection. Assessing the efficacy of different phages and phage-based products can be a complicated and is, using conventional microbiology techniques, a work-intensive task. The most commonly used technique to evaluate phage susceptibility is plaque assays, for example the double layer agar where lysis area (plaque) is clearly visible in the host lawn [25]. With this method, results can be available after 18 to 24 h, making it inapplicable for critically ill patients.

Here, we propose to use isothermal microcalorimetry as a rapid and less demanding alternative method to plaque assay. Isothermal microcalorimetry can measure microbial growth in many conditions [26,27,28]. The heat production rate (heatflow or thermal power) is considered as a proxy for the microbial metabolic activity. When the heat production rate is integrated over time, the resulting sigmoid heat curve is used as a conventional growth curve similar to those resulting from plate counts or turbidity measurements [26]. Still, isothermal microcalorimetry is much less applied for the study of viruses, in particular, the study of the interactions between phages and their target bacteria [29,30,31,32,33], was assessed only in conventional rich media. This study investigated the effect of a commercial polyvalent phage product on the growth of two urinary tract pathogens directly in urine and artificial urine.

## 2. Materials and Methods

### 2.1. Urine and Artificial Urine Preparation

Urine samples from three healthy donors were pooled and centrifuged shortly after to remove sediments, if present. After centrifugation, the urine was filtered through 0.22 µm pore size filters (Millipore, Stericup^®^, Billerica, MA, USA). Artificial urine was prepared by adding the following components: 10.0 g urea, 5.2 g NaCl, 2.1 g NaHCO_3_, 1.4 g Na_2_SO_4_, 1.3 g NH_4_Cl, 1.2 g K_2_HPO_4_, 1.0 g KH_2_PO_4_, 1.0 g peptone, 0.5 g MgSO_4_·7H_2_O, 0.4 g citric acid, 0.37 g CaCl_2_·2H_2_O, 0.1 g lactic acid, 70.0 mg creatinine, 10.0 mg FeSO_4_·7H_2_O, and 5.0 mg yeast extract to 1 L of deionized water. To enhance pathogen growth: 20 mg lactose, 20 mg saccharose, and 560 mg glucose were also added to the artificial urine solution. The artificial urine was sterilized by using a 0.22 µm pore size filter (Millipore, Stericup^®^, Billerica, MA, USA). The urine and artificial urine were then stored at 4 °C until use or at −80 °C for longer storage.

### 2.2. Microorganisms and Culture Conditions

Bacterial strains *E. coli* DSM 10142 and *P. mirabilis* DSM 4479 were used in this study. The two strains were stored in cryovials at −80 °C in 20% glycerol. Strains were grown at 37 °C overnight in Tryptic Soy Broth (17.0 g tryptone, 5.0 g NaCl, 3.0 g soy peptone, 2.5 g glucose, and 2.5 g K_2_HPO_4_ per 1 L purified water). Before use, the culture was diluted to a concentration of ca. 10^6^ colony forming units (CFU)·mL^−1^.

### 2.3. Phage Cocktail Description

The phage cocktail used in this study was an “E. coli-Proteus” bacteriophage solution (NPO Microgen, Nizhny Novgorod, Russia) obtained from an online pharmacy. The phage titer against *E. coli* DSM 10142 of this phage product was determined at 2.6 × 10^6^ ± 0.5 × 10^6^ Plaque forming units (PFU)·mL^−1^ by serial dilution and plating using the double-layer agar technique using Luria agar. This phage cocktail was previously characterized in metagenomic studies and consists of a mixture of 18 phages with higher numbers of T4-like and T7-like phages observed using transmission electron microscopy [34]. In addition, it was already used in human clinical trials [35].

### 2.4. Comparison of Isothermal Microcalorimetry with CFU, PFU and OD Data

To compare microcalorimetry data to conventional microbiological data, a measurement was performed with *E. coli* in artificial urine with or without the addition of phages. For the measurements, 5 mL of artificial urine inoculated at 10^7^ CFU·mL^−1^ were placed in 20 mL microcalorimeter vials and phage were added at 0.5% phage cocktail concentration (that is 10^4^ PFU·mL^−1^ phage) when required. Sterility controls were performed using uninoculated artificial urine. For the calorimetry measurements, vials were then sealed and introduced into the microcalorimeter previously set at 37 °C and equilibrated at this temperature for at least 2 days. After sample introduction and thermal equilibration, the heat flow corresponding to the microbial metabolic heat production rate was measured until it returned to the baseline.

For conventional microbiological measurements, 36 additional vials per condition were prepared in calorimetric vials and incubated at 37 °C in a separated incubator. CFU and optical density (OD) were measured over time by sacrificing 2 vials for each treatment. CFU were determined by serial dilution and plating on Luria agar. OD was read on an LLG-uniSPEC 4 spectrophotometer at 600 nm. Samples for phage determination were filtered using a 0.22 µm pore size syringe filter and kept in the fridge at 4 °C for no more than 24 h. After storage, the phage concentration was determined by serial dilution and plating using the double-layer agar technique with Luria agar. The experiment was repeated twice.

### 2.5. Phage Cocktail Efficacy

Both strains were tested against the polyvalent phage preparation described above [34,35]. For the test, sterile 4 mL calorimetry glass ampoules were filled with 3 mL of inoculated solution (urine or artificial urine). Phages were added at 1% (*v*/*v*-30 µL/vial) and 5% (*v*/*v*-150 µL/vial) of original phage cocktail. Untreated samples (no phage added) were used as controls and sterile medium as negative control. All samples were prepared in quadruplicates, except sterility controls, which were performed in triplicates.

The ampoules were then sealed using metal lid with a silicon rubber seal and introduced into the microcalorimeter previously set at 37 °C and equilibrated at this temperature for at least 2 days. After sample introduction and thermal equilibration, the heat flow corresponding to the microbial metabolic heat production rate was recorded for at least 96 h or until it returned to the baseline.

### 2.6. Combination with Trimethoprim/Sulfamethoxazole

In the case of *P. mirabilis*, growth was strongly reduced but not completely suppressed (see Result Section for details). For *P. mirabilis*, we investigated the combination of the phage cocktail with Trimethoprim/Sulfamethoxazole (TMP/SMX) (Nopil^®^ forte, Mepha Schweiz AG, Basel, Switzerland). A diluted overnight culture was added with 1% (*v*/*v*-30 µL/vial) of phages in combination with increasing concentrations of TMP/SMX. The following solutions were prepared: 1% phages + 50% minimal inhibitory concentration (MIC) TMP/SMX (4 mg·L^−1^), 1% phages + 25% MIC TMP/SMX (2 mg·L^−1^), and finally, 1% phages + 12.5% MIC TMP/SMX (1 mg·L^−1^). TMP/SMX (50% MIC) only, phages (1% *v*/*v*) only, and untreated cultures were used as controls. Uninoculated medium served as sterility control. All samples were prepared in quadruplicates except sterility controls that were done in triplicates.

### 2.7. Data Analysis

The raw microcalorimetric data were translated into usable microbiological data as growth rate (µ), lag phase duration (λ), and maximum growth (i.e., maximum heat produced-Q). To determine these parameters the integration of the heat flow data was performed, and the resulting heat curve was used as a proxy for the growth curve. The heat curve was fitted with the Gompertz growth model and the growth parameters were calculated. The data extraction and conversion to a CSV file was accomplished with TAM Assistant (TA Instruments, New Castle, DE, USA). All the remaining calculations and curve fittings were performed with the R version 3.6.3 [36] and the grofit package [37].

## 3. Results

### 3.1. Comparison of Isothermal Microcalorimetry and Conventional Microbiology

**Figure 1:**
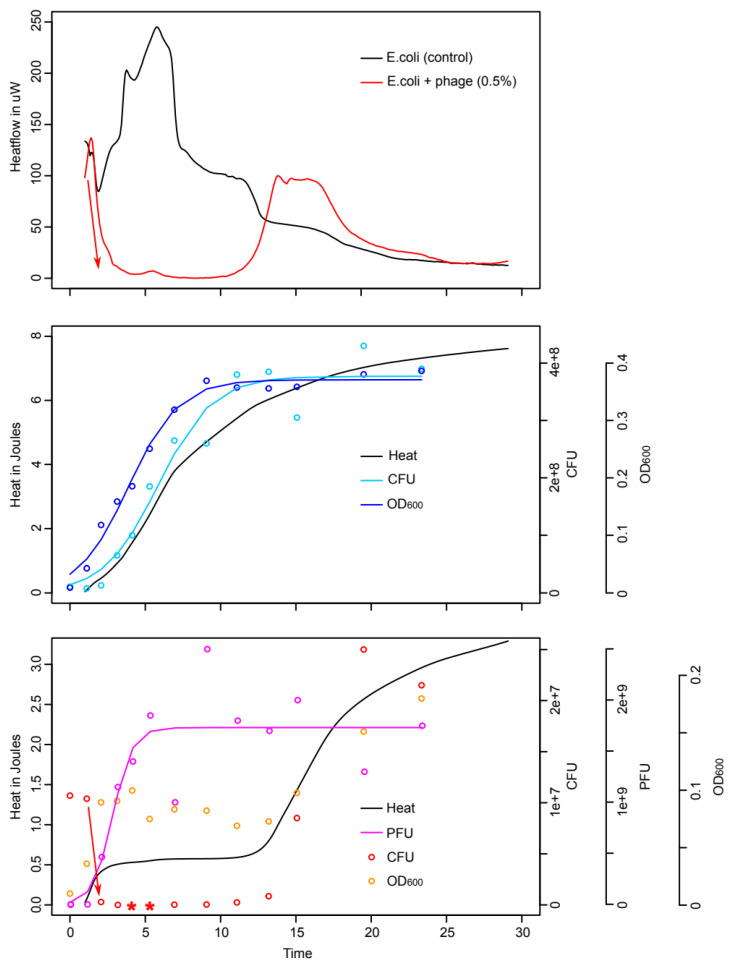
Growth of *E. coli* in artificial urine and with the addition of 0.5% *v/v* of phage cocktail (“E. coli-Proteus” bacteriophage solution, NPO Microgen, Russia). (**Top**) Heat flow data (raw data); (**Middle**) Heat over time data with matching CFU and OD_600_ data for the culture without phages. (**Bottom**) Heat over time data with matching CFU, PFU, and OD_600_ data. For CFU, PFU, and OD, points represent the raw measured data and the lines the fitted logistic model using those data. The red arrows indicate the heat flow decrease and the matching CFU decrease. * indicates value detection limit (below 100 CFU·mL^−1^). CFU = Colony Forming Unit, OD_600_ = Optical Density at 600 nm, PFU = Plaque Forming Units.

For *E. coli* cultures in artificial urine without the addition of the phage cocktail. the metabolic activity (i.e., the heat flow) rapidly rose and slowly decreased. When integrating the heat flow to obtain the heat over time curve (comparable to a growth curve), the growth pattern showed an s-shaped curved typically expected from bacterial growth. The heat over time curve for untreated *E. coli* fits well with the CFU and the OD data obtained from the parallel samples (Figure 1).

When the phage cocktail is added to *E. coli* in artificial urine, the metabolic activity rapidly decreases and returns to very low values (close to baseline) within 4 h. This matches well with the CFU counts performed that also show a strong decrease within the first hours. After 5 and 6 h the *E. coli* count was below detection limit (i.e., <100 CFU·mL^−1^). On the contrary, the OD remained at stable value but showed only a minimal decrease over time (Figure 1). In artificial urine, this might be due to the formation of struvite or other phosphate minerals that may nucleate on cell or cell debris, even in organisms that do not produce urease [38,39,40,41]. Indeed, microscopic investigation showed crystals in the artificial urine after culture with or without phages, thus, in such media, OD might overestimate the cell number and hinder the detection of lysis.

### 3.2. Monitoring of Phage Cocktail Antibacterial Activity

**Figure 2:**
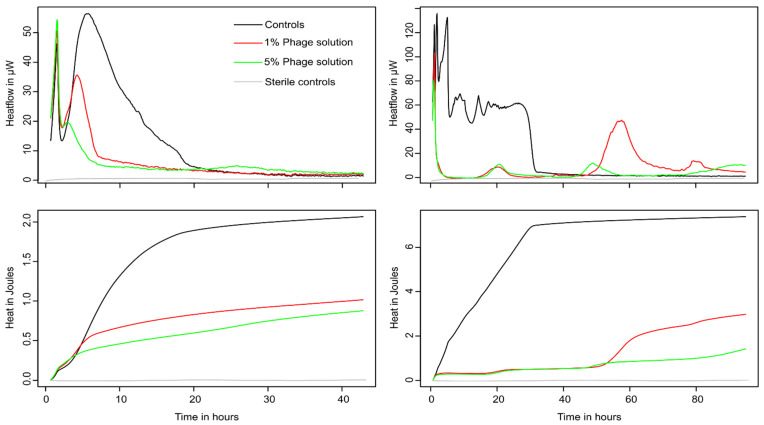
Representative curve showing the heat flow and integrated heat over time of *E. coli* exposed to increasing concentration of phage cocktail. (**Left**) Heat flow pattern in sterile filtered urine (**top**) and associated heat over time curve (**bottom**). (**Right**) Heat flow pattern in sterile artificial urine (**top**) and associated heat over time curve (**bottom**). Sterile controls are indicated in grey.

**Figure 3:**
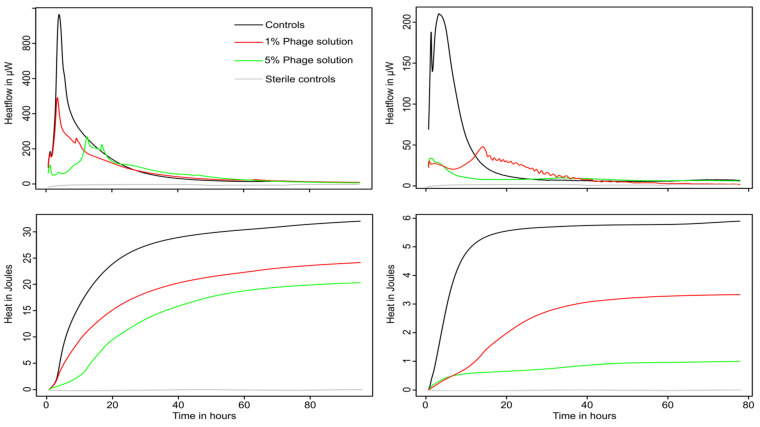
Representative curve showing the heat flow and integrated heat over time of *P. mirabilis* exposed to increasing concentration of phage cocktail. (**Left**) Heat flow pattern in sterile filtered urine (**top**) and associated heat over time curve (**bottom**). (**Right**) Heat flow pattern in sterile artificial urine (**top**) and associated heat over time curve (**bottom**). Sterile controls are indicated in grey.

Both bacterial strains were able to grow in both urine and artificial urine, as indicated by the detection of metabolic heat production (Figure 2 and Figure 3). Increasing concentrations of phage cocktail resulted in a strong reduction in the growth of both pathogens, marked by a rapid decrease in metabolic activity. The decrease in growth was dose-dependent, as shown by the variations in growth parameters (μ, λ, Q). In both cases, a decrease in the growth rate μ and the heat produced Q (Table 1 and Table 2) was observed demonstrating that bacterial growth was negatively affected by the phages. At higher phage concentration (5% *v*/*v*), a marked increase in the lag phase was detected only for *P. mirabilis*. An increase in the lag was also visible for *E. coli* but only in artificial urine and in the context of regrowth (see later sections). The heat production of *P. mirabilis* was much higher compared to *E. coli;* this was expected due to the presence of active urease (urea hydrolysis is an exothermic process with a ΔH of 119.2 kJ·mol^−1^). One main difference between the two pathogen was that *E. coli* activity was rapidly brought to baseline where no metabolic activity could be measured anymore following the addition of phages. However, regrowth was observed in artificial urine at several time points, meaning that some *E. coli* survived (note that the detection limit of the instrument used is ca 30,000 *E. coli*·mL^−1^). This regrowth pattern was also observed in previous studies (especially at lower initial phage concentration) with *E. coli* and other pathogens [30,42]. On the contrary, although the phage preparation strongly decreased the activity of *P. mirabilis* (>50% decrease in growth rate in urine and >80% decrease in growth rate in artificial urine compared to the controls), the initial growth was not fully suppressed (Figure 2).

The specific case of *E. coli* in artificial urine is of interest (Figure 2). Under these conditions, several peaks can be seen in the heat flow pattern after the initial return to the baseline induced by the phages. This indicates that *E. coli* metabolism was detected and suggests a regrowth of this pathogen. The pattern suggests a possible prey–predator interaction where some surviving *E. coli* might regrow until being re-infected by the phages.

### 3.3. Combination of Phages and Trimethoprim/Sulfamethoxazole

**Figure 4:**
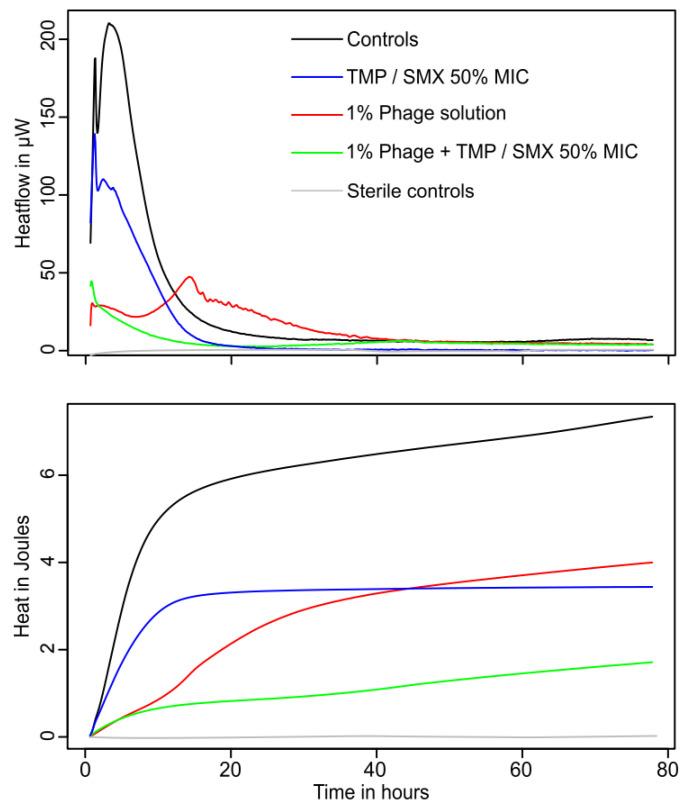
Representative curve showing the growth of *P. mirabilis* in sterile artificial urine when exposed to increasing 50% MIC TMP/SMX and/or 1% *v/v* phage cocktail (“E. coli-Proteus” bacteriophage solution, NPO Microgen, Nizhny Novgorod, Russia). (**Top**) Heat flow data; (**bottom**) integrated heat over time data (proxy for the growth curve). Sterile controls are indicated in grey. TMP/SMX = trimethoprim/sulfamethoxazole, MIC = minimal inhibitory concentration.

Neither phage solution alone nor TMP/SMX at 50% MIC succeeded in fully suppressing *P. mirabilis* growth, although a strong reduction in growth was observed (Figure 3). The heat flow pattern of the samples treated only with TMP/SMX generated a sharp peak with a shape comparable to the control but with decreased overall activity (i.e., heat flow). On the contrary, phage cocktail concentrations greater than 1% resulted in a smaller and broader peak reaching its maximum activity with a delay of roughly 10 h compared to the controls. When both phage and TMP/SMX were added together, the initial metabolic activity peak was rather low. In addition, the activity followed a decrease comparable to an exponential decay until activity returned to the baseline. In this case, no late peak or regrowth was observed, thus suggesting that the combination of TMP/SMX and phage succeeded in completely eradicating the pathogen. The growth parameters are shown in Table 3 and Figure 4.

## 4. Discussion

UTIs are often a reason to prescribe antibiotics and inevitably lead to an increase in antimicrobial resistance and the spread of multi-resistant bacteria [5,10]. Phage therapy used as an antibacterial treatment is thus of medical and economic interest [16,20,43]. Although standardized and marketed products are not available in most of the world, the current interest in phages suggest that such therapy may become more widespread. In this study, using isothermal microcalorimetry, we showed that the commercial phage product used induced bacterial lysis for *E. coli* and *P. mirabilis* in both types of urine. Our data are consistent with previous measures performed with *E. coli* B and the T3 and T4 phages in LB medium [30,31]. In addition, we also showed that the phage preparation used was compatible with additional TMP/SMX treatment. Isothermal microcalorimetry being a very sensitive real-time technique, it was also possible to see the speed of action and the potential microbial regrowth. Therefore, using isothermal microcalorimetry, one rapidly assesses which phage preparation could be of interest to treat a patient.

“Phage cocktails” containing multiple phage types have been used to counteract the narrow host range of single phages and to control the growth of *E. coli* infectious strains [6,44]. However, focusing on personalized phage therapy is possible as well. Such a strategy relies on the isolation of the bacterial pathogen from the infection site and further testing it against phages from a biobank. After this screening step, selected phages could be transmitted to the patient to rapidly treat the UTI [23,45]. Considering the different studies on rapid drug susceptibility testing using isothermal microcalorimetry [46] and previous studies with phages and viruses [29,30,31,32], we believe that providing a phagogram or a combined phagogram/antibiogram within 5 to 8 h is possible using patient urine directly, as UTI are often characterized by a high pathogen load with only rare polymicrobial infections. This would be of great interest, as it is expected to prevent the risk of mutant appearance [47,48,49]. In order to further speed up the process, isothermal microcalorimetry could be combined with a time-of-flight mass spectrometer MALDI-TOF [46,50,51] for preliminary identification of the pathogen and thus narrowing the range of phage product to be tested. Recent studies have shown that in acute UTI the MALDI-TOF identification could be performed with urine directly as well.

For isothermal microcalorimetry to become useful in this context, a defined and reproducible procedure is required. To increase reproducibility, artificial urine seems to be promising; still its use needs to be further discussed. Indeed, the addition of phages in urine resulted in a decrease in microbial activity that ultimately returned to baseline indicating a complete suppression of growth. However, in artificial urine two to three peaks could be detected after the initial return to baseline. In human urine, the presence of antibacterial enzymes/peptides, for example, α- and β-defensins or cathelicidin, probably contributes to inhibit the growth of *E. coli* and potentially *P. mirabilis* [52]. In addition, in artificial urine, the different peaks interpreted as regrowth of the pathogens seems to indicate a prey–predator behavior of the phages–host system [53]. Thus, there is a possibility that phage efficacy might be slightly underestimated in artificial urine. In real world scenarios, it is expected that if the growth rate is sufficiently reduced the surviving pathogens (if any) will ultimately be flushed out of the bladder by urination. Therefore, we assume that such approach would be safe for the patients.

Finally, we must recognize the limitation of the present study. Isothermal microcalorimeters do not allow shaking thus potentially limiting the contact between phages and host cells especially at low phage concentration. This might also have allowed for some regrowth observed. In addition, isothermal microcalorimetry measures active metabolism (i.e., active bacteria) but only provides an indirect picture of the phage activity as phage are using the microbial enzymatic machinery to replicate. However, considering the amount of work required for assessing bacteria and phage concentrations over time isothermal microcalorimetry remains a valuable proxy that strongly decreases the workload compared to CFU and PFU counts, OD or metabolic assays. Similarly, this study has focused on two strains only, thus making any generalization of the results too preliminary. Therefore, further studies should include more clinically relevant strains obtained from several patients. Further improvement could also include the optimization of the artificial urine composition. Indeed, artificial urine can probably be added with vitamin supplements to further improve the growth of uropathogens such as *E. coli* (in our study, *P. mirabilis* grew very well in artificial urine) and make the assay even faster. Finally, with respect to *P. mirabilis*, we must note that this pathogen is more commonly encountered in catheter-associated UTIs, where it often forms biofilms on catheters. Therefore, future studies should include antibiofilm assessment of the phage products. Similar assessment is possible and was performed for single phage already [30].

## 5. Conclusions

Isothermal microcalorimetry has proven to be a valuable technique when investigating the efficacy of phage preparation against various uropathogens in vitro. We foresee that isothermal microcalorimetry could be used to obtain rapid phagograms to choose the best phage or phage cocktail in the case of a urinary tract infection. The ease of measurement and the low workload combined to fast result delivery makes this technique appealing for future clinical studies. Alternatively, for the same reasons, isothermal microcalorimetry could also be used as a quality control tool for phage preparations, thus decreasing the workload for QA/QC control of such products in industrial settings.

## Figures and Tables

**Table 1:** Growth parameters of *E. coli* and *P. mirabilis* in urine when exposed to increasing amount of phage cocktail (“E. coli-Proteus” bacteriophage solution, NPO Microgen, Nizhny Novgorod, Russia). µ = growth rate, λ = lag phase duration, Q = maximum growth (i.e., maximum heat produced), TTP = Time-to peak.

*E. coli*				
Sample	μ (h^−1^)	λ (h)	Q (J)	TTP (h)
**Control**	0.18 ± 0.01	2.08 ± 0.18	1.93 ± 0.11	5.67 ± 0.12
**1%**	0.08 ± 0.01	0.00 ± 0.16	0.89 ± 0.16	1.50 ± 0.00
**5%**	0.04 ± 0.01	0.00 ± 2.69	0.57 ± 0.02	1.52 ± 0.04
***P. mirabilis***				
**Sample**	**μ (h^−1^)**	**λ (h)**	**Q (J)**	**TTP (h)**
**Control**	1.28 ± 0.05	0.0 ± 0.3	28.89 ± 0.3	4.00 ± 0.3
**1%**	0.66 ± 0.00	0.0 ± 0.0	21.09 ± 0.1	3.41 ± 0.0
**5%**	0.59 ± 0.08	4.6 ± 0.4	19.6 ± 1.6	12.5 ± 0.2

**Table 2:** Growth parameters of *E. coli* and *P. mirabilis* in artificial urine when exposed to increasing amount of phage cocktail (“E. coli-Proteus” bacteriophage solution, NPO Microgen, Nizhny Novgorod, Russia). µ = growth rate, λ = lag phase duration, Q = maximum growth (i.e., maximum heat produced).

*E. coli*	First Peak			Second Peak			Third Peak		
Sample	μ (h^−1^)	λ (h)	Q (J)	μ (h^−1^)	λ (h)	Q (J)	μ (h^−1^)	λ (h)	Q (J)
**Control**	0.29 ± 0.01	0.9 ± 0.2	7.0 ± 0.2						
**1%**	0.04 ± 0.02	17.8 ± 3.6	0.7 ± 0.5	0.09 ± 0.04	63.4 ± 12.1	1.6 ± 1.0			
**5%**	0.02 ± 0.01	18.3 ± 2.6	0.2 ± 0.1	0.03 ± 0.03	48.3 ± 32.1	0.5 ± 0.3	0.03	60.5	0.6
***P. mirabilis***									
**Sample**	**μ (h^−1^)**	**λ (h)**	**Q (J)**	**μ (h^−1^)**	**λ (h)**	**Q (J)**	**μ (h^−1^)**	**λ (h)**	**Q (J)**
**Control**	0.62 ± 0.04	1.1 ± 0.5	5.3 ± 0.5						
**1%**	0.11 ± 0.01	4.0 ± 1.1	2.9 ± 0.2						
**5%**	0.04 ± 0.02	0.0 ± 7.1	1.3 ± 0.0						

**Table 3:** Growth parameters of *P. mirabilis* in artificial urine when exposed to 1% (*v*/*v*) phage cocktail (“E. coli-Proteus” bacteriophage solution, NPO Microgen, Nizhny Novgorod Russia) and added with increasing concentrations of TMP/SMX. µ = growth rate, λ = lag phase duration, Q = maximum growth (i.e., maximum heat produced), TTP = Time to peak, TMP/SMX = trimethoprim/sulfamethoxazole, MIC = minimal inhibitory concentration.

*P. mirabilis* + TMP/SMX				
Sample	μ (h^−1^)	λ (h)	Q (J)	TTP (h)
**Control**	0.54 ± 0.01	0.0 ± 0.0	5.2 ± 0.1	3.2 ± 0.1
**1% phage**	0.11 ± 0.01	4.0 ± 1.1	2.9 ± 0.2	14.2 ± 0.1
**1% phage + 50% MIC**	0.01 ± 0.01	0.0 ± 13.6	1.0 ± 0.7	0.6 ± 0.4
**1% phage +25% MIC**	0.01 ± 0.01	0.0 ± 11.9	0.8 ± 0.7	0.7 ± 0.5
**TMP/SMX: 50% MIC**	0.31 ± 0.01	0.0 ± 0.2	3.6 ± 0.1	1.3 ± 0.0
**Blanks**	0.00 ± 0.00	0.00 ± 0.00	0.00 ± 0.00	0.00 ± 0.00

## Data Availability

Data presented in this study are available on request from the corresponding author.
